# Quel diagnostic devant une éruption fébrile?

**DOI:** 10.11604/pamj.2017.27.227.12657

**Published:** 2017-07-28

**Authors:** Nadia Fihmi, Imane Alouani, Abdelhafid Elmrahi, Nada Zizi, Siham Dikhaye

**Affiliations:** 1 Service de Dermatologie, CHU Mohammed VI d'Oujda, Faculté de Médecine et Pharmacie, Université Mohammed Premier d'Oujda, Maroc

**Keywords:** Exanthème, fièvre, toxines, diagnostic précoce, Exanthema, fever, toxins, early diagnosis

## Abstract

Le patient avec exanthème fébrile pose au médecin de premier recours un véritable défi diagnostic. Nous rapportons un cas original d'exanthème maculeux fébrile que nous avons pu rattacher à un sepsis secondaire à une pelvipéritonite et cholécystite aigue. L'anamnèse, l'examen clinique de qualité, les examens paracliniques et l'évolution favorable nous a permis de retenir l'origine infectieuse de l'exanthème, sans avoir de confirmation microbiologique dans notre observation. La peau est un excellent marqueur de l'infection. Les signes cutanés figurent en effet parmi les symptômes le plus souvent observés à la phase initiale d'une état septique. La lesion la plus fréquente est l'exanthème, éruption cutanée liée aux effets systémiques d'une micro-organisme sur la peau. Le diagnostic précoce peut prévenir les complications d'une infection inapparente.

## Abstract (en)

The patient with febrile rash poses a real diagnostic challenge to primary care physician. We report an original case of febrile macular rash whose etiology was related to sepsis secondary to pelviperitonitis and acute cholecystitis. Patient's history, careful physical examination, paraclinical examinations and favorable outcome allowed to retain the infectious origin of the rash, without microbiological confirmation in our study. The skin is an excellent marker for infection. Cutaneous manifestations are the most common signs observed in patients with sepsis at an early stage. Exanthema is the most common lesion; it is due to systemic effects of a microorganism infecting the skin. If there are no clinical signs of infection, early diagnosis can prevent complications.

## Introduction

Le patient avec exanthème fébrile pose au médecin de premier recours un véritable défi diagnostic. La difficulté de leur prise en charge tient au grand nombre de causes possibles et à leur intrication fréquente chez un même malade. Nous rapportons un cas original d'exanthème maculeux fébrile dû à un sepsis secondaire à une pelvipéritonite et cholécystite aigue

## Patient et observation

**Figure 1:**
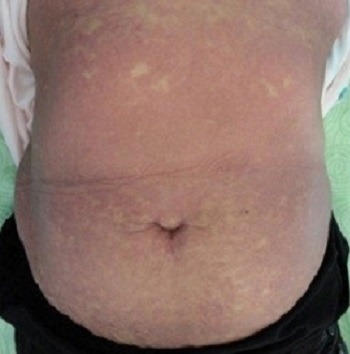
Erythème diffus scarlatiniforme

**Figure 2:**
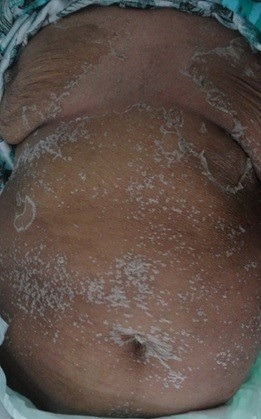
Desquamation généralisée en lambeau

Une femme de 50 ans hémodialysée chronique est hospitalisée pour un exanthème maculeux prurigineux fébrile évoluant depuis cinq jours précédé deux jours avant par une symptomatologie digestive faite de douleurs abdominales et diarrhées liquidiennes, le tout évoluant dans un contexte de fièvre et d'altération de l'état général. L'interrogatoire retrouvait la notion de prise médicamenteuse d'inhibiteur de la pompe à proton (Esoméprazol) trois semaines avant l'éruption. A l'examen clinique la patiente était fébrile à 38.5°C, tachycarde à 120 battements/minute et normotendue. Elle présentait un érythème diffus scarlatiniforme (Figure 1) avec des lésions purpuriques au niveau des membres inférieurs. On observait une légère hyperhémie conjonctivale, un énanthème buccal et vulvaire et des leucorrhées blanchâtres nauséabondes avec une douleur à la mobilisation du col utérin. Par ailleurs la patiente présentait des polyadénopathies et sensibilité d'hypochondre droit sans hépatosplénomégalie. Devant ce tableau clinique nous avons évoqué: une toxidermie médicamenteuse, un exanthème toxinique et exanthème viral. Les examens biologiques révélaient un syndrome inflammatoire majeur (C-reactive protein: 210 mg/l, fibrinogène: 5.20 g/l). L'hémogramme montrait une hyperleucocytose (13000 éléments /mm^3^) avec polynucléaires neutrophiles (PNN: 10400 éléments /mm^3^) et hyperéosinophilie (PNE: 1130 éléments /mm^3^). La Procalcitonine était très augmentée à 26 ng/l. Le bilan infectieux trouvait une amibiase à la coproparasitologie des selles. Les sérologies hépatitiques B et C, syphilitique et VIH ainsi que les hémocultures étaient négatives. L'échographie abdominale et endovaginale révélait une cholécystite aigue et une pelvipéritonite sur pyosalpinx respectivement. Il n'y avait pas d'anomalie à l'échographie transthoracique. À la lumière de l'examen clinique et des investigations paracliniques, le diagnostic final retenu était celui d'une exanthème toxinique révélateur d'une sepsis sur pelvipéritonite et cholécystite aigue. Un transfert en réanimation était alors nécessaire devant l'aggravation du tableau clinique et un traitement médical conservateur par antibiothérapie et antifongique par voie intraveineuse était indiqué vu le risque opératoire très élevé. Une amélioration clinique et biologique était rapidement observée après 10 jours de traitement médical avec disparition de la fièvre, desquamation généralisée en lambeaux (Figure 2), diminution de la CRP et normalisation de l'hémogramme.

## Discussion

**Figure 3:**
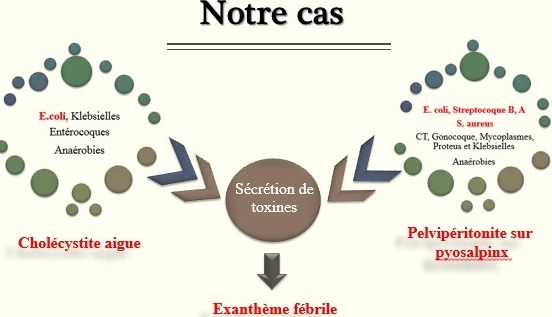
Les germes les plus incriminés et reconnues dans la littérature comme sécréteur de toxines

La peau est un excellent marqueur de l'infection. Les signes cutanés figurent en effet parmi les symptômes le plus souvent observés à la phase initiale d'une état septique. La lésion la plus fréquente est l'exanthème lié aux effets systémiques des micro-organismes sur la peau. Les principales causes des éruptions aiguës généralisées fébriles chez l'adulte sont dominées par sont les cause médicamenteuses, les infections virales et les éruptions toxiniques [1, 2]. Dans la série de Bialecki d'exanthème fébrile sur 100 patients, 50% des cas étaient d'origine infectieuse [3]. On distingue schématiquement deux types de syndromes cliniques selon que les symptômes sont liés à l'action directe de la bactérie ou à l'action de toxines [4]. L'agressivité thérapeutique durant les premières heures est probablement la thérapeutique la plus efficace pour réduire la mortalité liée au sepsis qui constitue la première cause de décès en réanimation [5]. L'anamnèse, l'examen clinique de qualité, les examens paracliniques et l'évolution favorable nous a permis de retenir l'origine infectieuse de l'exanthème, sans avoir de confirmation microbiologique dans notre observation. Notre patiente présentait sepsis secondaire à une cholécystite aigue et pelvipéritonite dont les germes (Figure 3) les plus souvent incriminés sont reconnues dans la littérature comme sécréteur de toxines [6, 7]. Celles-ci sont à l'origine de l'exanthème fébrile dans notre cas.

## Conclusion

Cette observation illustre bien l'importance des manifestations cutanées dans les infections bactériennes, virales ou parasitaires. Leur constatation doit faire rechercher un état septique même en l'absence d'autres signes évocateurs. Le diagnostic d'une exanthème fébrile requiert un interrogatoire et un examen clinique minutieux et complet afin de ne pas laisser évoluer une infection inapparente.

## Conflits d’intérêts

Les auteurs ne déclarent aucun conflit d'intérêts.
